# Supplementary material for: Modeling glioblastoma heterogeneity as a dynamic network of cell states
Source: Mol Syst Biol. 2021 Sep 16;17(9):e10105. doi: 10.15252/msb.202010105 (PMC8444284; doi:10.15252/msb.202010105)
Supplement: Supplementary file 5 — Source Data for Figure 3 [file MSB-17-e10105-s001.zip › Figure3A_sourcedata/GSEA_3065/nosigdb_state2.GseaPreranked.1622718419215/RICHARDS_ZHONG_NATURE_2018_ASTROCYTES_UPREG.html]

Details for gene set RICHARDS\_ZHONG\_NATURE\_2018\_ASTROCYTES\_UPREG[GSEA]

|  || Dataset | state2 |
| Phenotype | NoPhenotypeAvailable |
| Upregulated in class | na\_neg |
| GeneSet | RICHARDS\_ZHONG\_NATURE\_2018\_ASTROCYTES\_UPREG |
| Enrichment Score (ES) | -0.5521537 |
| Normalized Enrichment Score (NES) | -2.3582706 |
| Nominal p-value | 0.0 |
| FDR q-value | 0.0 |
| FWER p-Value | 0.0 |
Table: GSEA Results Summary

  

Fig 1: Enrichment plot: RICHARDS\_ZHONG\_NATURE\_2018\_ASTROCYTES\_UPREG      
 Profile of the Running ES Score & Positions of GeneSet Members on the Rank Ordered List

  

| PROBE | GENE SYMBOL | GENE\_TITLE | RANK IN GENE LIST | RANK METRIC SCORE | RUNNING ES | CORE ENRICHMENT || 1 | DBI |  |  | 251 | 0.252 | -0.0167 | No |
| 2 | PHGDH |  |  | 271 | 0.243 | -0.0086 | No |
| 3 | GNG5 |  |  | 389 | 0.207 | -0.0127 | No |
| 4 | AIF1L |  |  | 481 | 0.183 | -0.0149 | No |
| 5 | PSAT1 |  |  | 561 | 0.170 | -0.0163 | No |
| 6 | TRIB2 |  |  | 636 | 0.158 | -0.0178 | No |
| 7 | S100B |  |  | 688 | 0.151 | -0.0170 | No |
| 8 | FAM181B |  |  | 696 | 0.151 | -0.0114 | No |
| 9 | GNG11 |  |  | 698 | 0.151 | -0.0053 | No |
| 10 | ID4 |  |  | 705 | 0.150 | 0.0004 | No |
| 11 | MT2A |  |  | 731 | 0.146 | 0.0038 | No |
| 12 | FABP5 |  |  | 796 | 0.138 | 0.0026 | No |
| 13 | FABP7 |  |  | 810 | 0.137 | 0.0069 | No |
| 14 | SNX3 |  |  | 823 | 0.136 | 0.0113 | No |
| 15 | SPRY2 |  |  | 870 | 0.131 | 0.0118 | No |
| 16 | IDI1 |  |  | 878 | 0.130 | 0.0164 | No |
| 17 | PREX1 |  |  | 890 | 0.129 | 0.0207 | No |
| 18 | SNX5 |  |  | 942 | 0.125 | 0.0203 | No |
| 19 | SOX8 |  |  | 945 | 0.124 | 0.0253 | No |
| 20 | SOX2 |  |  | 1036 | 0.117 | 0.0204 | No |
| 21 | APBB2 |  |  | 1130 | 0.110 | 0.0149 | No |
| 22 | GNG12 |  |  | 1154 | 0.108 | 0.0169 | No |
| 23 | DDAH1 |  |  | 1260 | 0.101 | 0.0097 | No |
| 24 | SOX9 |  |  | 1466 | 0.088 | -0.0088 | No |
| 25 | DCXR |  |  | 1477 | 0.088 | -0.0062 | No |
| 26 | WSCD1 |  |  | 1616 | 0.081 | -0.0178 | No |
| 27 | SPRY1 |  |  | 1623 | 0.081 | -0.0151 | No |
| 28 | LITAF |  |  | 1685 | 0.078 | -0.0185 | No |
| 29 | CBR1 |  |  | 1744 | 0.075 | -0.0217 | No |
| 30 | JUN |  |  | 1805 | 0.072 | -0.0252 | No |
| 31 | PDLIM3 |  |  | 1841 | 0.071 | -0.0260 | No |
| 32 | ETV5 |  |  | 1843 | 0.071 | -0.0232 | No |
| 33 | C3orf70 |  |  | 1976 | 0.065 | -0.0348 | No |
| 34 | RAB31 |  |  | 1977 | 0.065 | -0.0320 | No |
| 35 | RAB34 |  |  | 2057 | 0.062 | -0.0380 | No |
| 36 | PEA15 |  |  | 2161 | 0.059 | -0.0467 | No |
| 37 | RHOC |  |  | 2261 | 0.055 | -0.0552 | No |
| 38 | MGLL |  |  | 2293 | 0.054 | -0.0563 | No |
| 39 | ZFP36L1 |  |  | 2320 | 0.053 | -0.0569 | No |
| 40 | CNN3 |  |  | 2349 | 0.052 | -0.0577 | No |
| 41 | ADD3 |  |  | 2401 | 0.051 | -0.0612 | No |
| 42 | MT3 |  |  | 2560 | 0.046 | -0.0764 | No |
| 43 | ARHGEF26 |  |  | 2624 | 0.044 | -0.0814 | No |
| 44 | DARS |  |  | 2795 | 0.039 | -0.0982 | No |
| 45 | ANXA5 |  |  | 2812 | 0.039 | -0.0984 | No |
| 46 | KLHDC8A |  |  | 2839 | 0.038 | -0.0996 | No |
| 47 | C1orf61 |  |  | 2879 | 0.037 | -0.1023 | No |
| 48 | ALDH6A1 |  |  | 2912 | 0.036 | -0.1042 | No |
| 49 | PLEKHB1 |  |  | 2927 | 0.036 | -0.1043 | No |
| 50 | GNG7 |  |  | 2967 | 0.035 | -0.1071 | No |
| 51 | CDC42EP1 |  |  | 3034 | 0.033 | -0.1128 | No |
| 52 | C1orf122 |  |  | 3057 | 0.033 | -0.1139 | No |
| 53 | EFHD2 |  |  | 3081 | 0.032 | -0.1150 | No |
| 54 | TSPAN3 |  |  | 3103 | 0.032 | -0.1160 | No |
| 55 | IFI27L1 |  |  | 3114 | 0.031 | -0.1158 | No |
| 56 | ZFP36L2 |  |  | 3122 | 0.031 | -0.1152 | No |
| 57 | RGCC |  |  | 3143 | 0.031 | -0.1161 | No |
| 58 | C2orf72 |  |  | 3151 | 0.031 | -0.1156 | No |
| 59 | PHYHIPL |  |  | 3290 | 0.028 | -0.1294 | No |
| 60 | HSPB1 |  |  | 3629 | 0.022 | -0.1652 | No |
| 61 | GLUD1 |  |  | 3694 | 0.021 | -0.1713 | No |
| 62 | ABHD3 |  |  | 3775 | 0.019 | -0.1792 | No |
| 63 | SLC9A3R1 |  |  | 3823 | 0.018 | -0.1835 | No |
| 64 | CRYL1 |  |  | 3830 | 0.018 | -0.1834 | No |
| 65 | UBL3 |  |  | 3891 | 0.017 | -0.1892 | No |
| 66 | ETV1 |  |  | 3908 | 0.017 | -0.1902 | No |
| 67 | FERMT2 |  |  | 3980 | 0.016 | -0.1973 | No |
| 68 | ZFYVE21 |  |  | 3999 | 0.015 | -0.1986 | No |
| 69 | OAT |  |  | 4004 | 0.015 | -0.1984 | No |
| 70 | SPATA6 |  |  | 4032 | 0.015 | -0.2007 | No |
| 71 | NDRG2 |  |  | 4043 | 0.015 | -0.2011 | No |
| 72 | SHISA4 |  |  | 4125 | 0.014 | -0.2094 | No |
| 73 | ELN |  |  | 4163 | 0.013 | -0.2128 | No |
| 74 | HMGCS1 |  |  | 4272 | 0.011 | -0.2241 | No |
| 75 | PHACTR2 |  |  | 4339 | 0.010 | -0.2309 | No |
| 76 | ENHO |  |  | 4360 | 0.010 | -0.2326 | No |
| 77 | P3H4 |  |  | 4382 | 0.009 | -0.2345 | No |
| 78 | TOR1AIP1 |  |  | 4475 | 0.008 | -0.2442 | No |
| 79 | BDH2 |  |  | 4538 | 0.007 | -0.2507 | No |
| 80 | OLFM2 |  |  | 4808 | 0.003 | -0.2798 | No |
| 81 | HADHB |  |  | 4810 | 0.003 | -0.2798 | No |
| 82 | ROBO3 |  |  | 4933 | 0.001 | -0.2930 | No |
| 83 | SASH1 |  |  | 4986 | 0.000 | -0.2986 | No |
| 84 | ABCD3 |  |  | 5091 | -0.001 | -0.3099 | No |
| 85 | GULP1 |  |  | 5158 | -0.003 | -0.3169 | No |
| 86 | TCF7L2 |  |  | 5199 | -0.003 | -0.3211 | No |
| 87 | GNPTG |  |  | 5284 | -0.005 | -0.3301 | No |
| 88 | CDC42EP4 |  |  | 5335 | -0.005 | -0.3353 | No |
| 89 | SLC38A3 |  |  | 5423 | -0.007 | -0.3444 | No |
| 90 | TSC22D4 |  |  | 5543 | -0.009 | -0.3570 | No |
| 91 | SIRPA |  |  | 5641 | -0.011 | -0.3671 | No |
| 92 | HAPLN3 |  |  | 5663 | -0.011 | -0.3689 | No |
| 93 | CCDC80 |  |  | 6077 | -0.018 | -0.4130 | No |
| 94 | PPP1R16A |  |  | 6146 | -0.019 | -0.4196 | No |
| 95 | HOPX |  |  | 6156 | -0.019 | -0.4197 | No |
| 96 | HES1 |  |  | 6191 | -0.020 | -0.4226 | No |
| 97 | CAMK2G |  |  | 6251 | -0.021 | -0.4281 | No |
| 98 | EMC2 |  |  | 6312 | -0.022 | -0.4337 | No |
| 99 | TSPAN7 |  |  | 6363 | -0.023 | -0.4382 | No |
| 100 | PDPN |  |  | 6446 | -0.024 | -0.4461 | No |
| 101 | SERPINB6 |  |  | 6465 | -0.025 | -0.4470 | No |
| 102 | NLGN3 |  |  | 6477 | -0.025 | -0.4472 | No |
| 103 | EEPD1 |  |  | 6558 | -0.026 | -0.4548 | No |
| 104 | ABAT |  |  | 6574 | -0.027 | -0.4553 | No |
| 105 | PAQR8 |  |  | 6579 | -0.027 | -0.4546 | No |
| 106 | ADORA2B |  |  | 6618 | -0.028 | -0.4575 | No |
| 107 | BBS2 |  |  | 6732 | -0.030 | -0.4686 | No |
| 108 | TECPR2 |  |  | 6733 | -0.030 | -0.4673 | No |
| 109 | MYO10 |  |  | 6758 | -0.031 | -0.4686 | No |
| 110 | EFEMP2 |  |  | 6895 | -0.034 | -0.4820 | No |
| 111 | GRB14 |  |  | 6973 | -0.036 | -0.4888 | No |
| 112 | ELOVL5 |  |  | 7020 | -0.037 | -0.4923 | No |
| 113 | LRP4 |  |  | 7084 | -0.039 | -0.4975 | No |
| 114 | NOTCH3 |  |  | 7196 | -0.042 | -0.5078 | No |
| 115 | TNFRSF19 |  |  | 7226 | -0.043 | -0.5092 | No |
| 116 | SLC1A4 |  |  | 7280 | -0.044 | -0.5131 | No |
| 117 | TIMP3 |  |  | 7309 | -0.045 | -0.5142 | No |
| 118 | CSPG5 |  |  | 7377 | -0.047 | -0.5196 | No |
| 119 | RAMP1 |  |  | 7435 | -0.048 | -0.5237 | No |
| 120 | CYP51A1 |  |  | 7467 | -0.050 | -0.5250 | No |
| 121 | QKI |  |  | 7478 | -0.050 | -0.5240 | No |
| 122 | ID1 |  |  | 7519 | -0.051 | -0.5262 | No |
| 123 | GPR137B |  |  | 7585 | -0.053 | -0.5311 | No |
| 124 | F3 |  |  | 7662 | -0.055 | -0.5370 | No |
| 125 | PDLIM5 |  |  | 7663 | -0.055 | -0.5348 | No |
| 126 | MAGT1 |  |  | 7724 | -0.057 | -0.5389 | No |
| 127 | SLC25A18 |  |  | 7731 | -0.057 | -0.5371 | No |
| 128 | SOAT1 |  |  | 7758 | -0.058 | -0.5375 | No |
| 129 | DNER |  |  | 7777 | -0.059 | -0.5370 | No |
| 130 | MEIS1 |  |  | 7785 | -0.059 | -0.5353 | No |
| 131 | HES4 |  |  | 7819 | -0.060 | -0.5364 | No |
| 132 | RASSF4 |  |  | 7851 | -0.061 | -0.5372 | No |
| 133 | GATM |  |  | 7990 | -0.067 | -0.5494 | Yes |
| 134 | YIF1A |  |  | 7991 | -0.067 | -0.5466 | Yes |
| 135 | TSPAN6 |  |  | 8000 | -0.067 | -0.5446 | Yes |
| 136 | CA12 |  |  | 8005 | -0.067 | -0.5422 | Yes |
| 137 | BCAP29 |  |  | 8007 | -0.068 | -0.5395 | Yes |
| 138 | SDC3 |  |  | 8064 | -0.070 | -0.5427 | Yes |
| 139 | TEX264 |  |  | 8074 | -0.070 | -0.5407 | Yes |
| 140 | S100A16 |  |  | 8127 | -0.073 | -0.5433 | Yes |
| 141 | LIFR |  |  | 8134 | -0.073 | -0.5409 | Yes |
| 142 | CD82 |  |  | 8151 | -0.074 | -0.5395 | Yes |
| 143 | LIPA |  |  | 8178 | -0.076 | -0.5392 | Yes |
| 144 | GDPD2 |  |  | 8201 | -0.077 | -0.5383 | Yes |
| 145 | AASS |  |  | 8209 | -0.078 | -0.5358 | Yes |
| 146 | RFX4 |  |  | 8211 | -0.078 | -0.5327 | Yes |
| 147 | GLUL |  |  | 8307 | -0.083 | -0.5395 | Yes |
| 148 | SFXN5 |  |  | 8309 | -0.083 | -0.5362 | Yes |
| 149 | PBXIP1 |  |  | 8337 | -0.085 | -0.5356 | Yes |
| 150 | NOTCH2 |  |  | 8358 | -0.086 | -0.5341 | Yes |
| 151 | SLC4A4 |  |  | 8359 | -0.086 | -0.5305 | Yes |
| 152 | P4HA1 |  |  | 8398 | -0.089 | -0.5309 | Yes |
| 153 | FADS2 |  |  | 8492 | -0.095 | -0.5371 | Yes |
| 154 | ATP1B2 |  |  | 8494 | -0.095 | -0.5332 | Yes |
| 155 | BMP7 |  |  | 8549 | -0.100 | -0.5349 | Yes |
| 156 | HEY1 |  |  | 8555 | -0.100 | -0.5312 | Yes |
| 157 | MFGE8 |  |  | 8573 | -0.101 | -0.5288 | Yes |
| 158 | SEMA6A |  |  | 8574 | -0.101 | -0.5246 | Yes |
| 159 | ITM2C |  |  | 8600 | -0.103 | -0.5230 | Yes |
| 160 | CTSL |  |  | 8637 | -0.106 | -0.5225 | Yes |
| 161 | G6PC3 |  |  | 8676 | -0.109 | -0.5220 | Yes |
| 162 | LGALS1 |  |  | 8680 | -0.110 | -0.5177 | Yes |
| 163 | VIM |  |  | 8691 | -0.110 | -0.5142 | Yes |
| 164 | LEPROT |  |  | 8698 | -0.111 | -0.5102 | Yes |
| 165 | CST3 |  |  | 8712 | -0.112 | -0.5070 | Yes |
| 166 | RGS20 |  |  | 8714 | -0.112 | -0.5024 | Yes |
| 167 | IFITM3 |  |  | 8720 | -0.113 | -0.4982 | Yes |
| 168 | GJA1 |  |  | 8728 | -0.114 | -0.4942 | Yes |
| 169 | TRIM47 |  |  | 8738 | -0.115 | -0.4904 | Yes |
| 170 | LRRN3 |  |  | 8753 | -0.117 | -0.4870 | Yes |
| 171 | BCHE |  |  | 8775 | -0.119 | -0.4843 | Yes |
| 172 | ID2 |  |  | 8781 | -0.120 | -0.4799 | Yes |
| 173 | RGMA |  |  | 8807 | -0.123 | -0.4774 | Yes |
| 174 | DTNA |  |  | 8828 | -0.126 | -0.4743 | Yes |
| 175 | PLTP |  |  | 8849 | -0.129 | -0.4711 | Yes |
| 176 | SEZ6L |  |  | 8858 | -0.130 | -0.4665 | Yes |
| 177 | TP53I3 |  |  | 8860 | -0.131 | -0.4611 | Yes |
| 178 | HSD17B12 |  |  | 8878 | -0.133 | -0.4574 | Yes |
| 179 | S100A13 |  |  | 8880 | -0.133 | -0.4520 | Yes |
| 180 | ITPR2 |  |  | 8925 | -0.138 | -0.4510 | Yes |
| 181 | RCN1 |  |  | 8929 | -0.138 | -0.4455 | Yes |
| 182 | GPRC5B |  |  | 8956 | -0.142 | -0.4424 | Yes |
| 183 | SCD |  |  | 8982 | -0.146 | -0.4390 | Yes |
| 184 | PTN |  |  | 8989 | -0.147 | -0.4335 | Yes |
| 185 | SEMA6D |  |  | 8994 | -0.148 | -0.4277 | Yes |
| 186 | DHCR7 |  |  | 9009 | -0.150 | -0.4230 | Yes |
| 187 | TTYH1 |  |  | 9029 | -0.154 | -0.4186 | Yes |
| 188 | PON2 |  |  | 9030 | -0.154 | -0.4122 | Yes |
| 189 | COMT |  |  | 9039 | -0.156 | -0.4065 | Yes |
| 190 | PLIN3 |  |  | 9043 | -0.157 | -0.4003 | Yes |
| 191 | SLC1A3 |  |  | 9052 | -0.158 | -0.3945 | Yes |
| 192 | LRRC17 |  |  | 9058 | -0.159 | -0.3884 | Yes |
| 193 | TFPI |  |  | 9060 | -0.159 | -0.3818 | Yes |
| 194 | MRC2 |  |  | 9106 | -0.167 | -0.3798 | Yes |
| 195 | LGALS3BP |  |  | 9114 | -0.169 | -0.3734 | Yes |
| 196 | ALCAM |  |  | 9132 | -0.173 | -0.3680 | Yes |
| 197 | PRCP |  |  | 9153 | -0.178 | -0.3628 | Yes |
| 198 | MSMO1 |  |  | 9162 | -0.179 | -0.3561 | Yes |
| 199 | HTRA1 |  |  | 9178 | -0.184 | -0.3501 | Yes |
| 200 | SYPL1 |  |  | 9182 | -0.185 | -0.3427 | Yes |
| 201 | SERPINE2 |  |  | 9186 | -0.186 | -0.3352 | Yes |
| 202 | DAG1 |  |  | 9192 | -0.187 | -0.3279 | Yes |
| 203 | SCRG1 |  |  | 9200 | -0.191 | -0.3207 | Yes |
| 204 | TMEM132B |  |  | 9205 | -0.192 | -0.3131 | Yes |
| 205 | FAT1 |  |  | 9207 | -0.193 | -0.3051 | Yes |
| 206 | NT5E |  |  | 9208 | -0.194 | -0.2970 | Yes |
| 207 | PLEC |  |  | 9220 | -0.200 | -0.2898 | Yes |
| 208 | LRIG1 |  |  | 9229 | -0.204 | -0.2822 | Yes |
| 209 | PTTG1IP |  |  | 9230 | -0.204 | -0.2736 | Yes |
| 210 | SLC3A2 |  |  | 9268 | -0.220 | -0.2684 | Yes |
| 211 | MLC1 |  |  | 9269 | -0.220 | -0.2592 | Yes |
| 212 | ADGRG1 |  |  | 9275 | -0.222 | -0.2505 | Yes |
| 213 | NTRK2 |  |  | 9299 | -0.230 | -0.2434 | Yes |
| 214 | LGALS3 |  |  | 9308 | -0.236 | -0.2344 | Yes |
| 215 | LAMB2 |  |  | 9319 | -0.246 | -0.2252 | Yes |
| 216 | CD9 |  |  | 9324 | -0.248 | -0.2152 | Yes |
| 217 | TM7SF2 |  |  | 9330 | -0.252 | -0.2052 | Yes |
| 218 | DDR1 |  |  | 9345 | -0.266 | -0.1956 | Yes |
| 219 | ITGAV |  |  | 9346 | -0.269 | -0.1844 | Yes |
| 220 | PMP22 |  |  | 9347 | -0.271 | -0.1730 | Yes |
| 221 | PTPRZ1 |  |  | 9351 | -0.272 | -0.1620 | Yes |
| 222 | BCAN |  |  | 9353 | -0.276 | -0.1505 | Yes |
| 223 | SPARCL1 |  |  | 9360 | -0.288 | -0.1391 | Yes |
| 224 | EDNRB |  |  | 9369 | -0.301 | -0.1274 | Yes |
| 225 | B2M |  |  | 9383 | -0.331 | -0.1150 | Yes |
| 226 | TIMP1 |  |  | 9387 | -0.334 | -0.1014 | Yes |
| 227 | GPM6B |  |  | 9389 | -0.344 | -0.0871 | Yes |
| 228 | CD63 |  |  | 9394 | -0.353 | -0.0727 | Yes |
| 229 | TNC |  |  | 9403 | -0.383 | -0.0576 | Yes |
| 230 | DKK3 |  |  | 9411 | -0.414 | -0.0410 | Yes |
| 231 | GFAP |  |  | 9425 | -0.504 | -0.0214 | Yes |
| 232 | CLU |  |  | 9430 | -0.547 | 0.0011 | Yes |
Table: GSEA details [plain text format]

  

Fig 2: RICHARDS\_ZHONG\_NATURE\_2018\_ASTROCYTES\_UPREG: Random ES distribution      
 Gene set null distribution of ES for **RICHARDS\_ZHONG\_NATURE\_2018\_ASTROCYTES\_UPREG**
